# Exploratory seminal plasma metabolomics and independent HPLC–UV evaluation in men with impaired semen quality

**DOI:** 10.3389/fendo.2026.1909718

**Published:** 2026-08-25

**Authors:** Jiale Xu, Li Guo, Lingxiao Xiong, Jun Li, Peiqiang Wu, Yanmin Ma, Juan Yang, Yuefeng Du

**Affiliations:** 1 Department of Urology, The First Affiliated Hospital of Xi’an Jiaotong University, Xi’an, Shaanxi, China; 2 Department of Reproductive Medicine, The First Affiliated Hospital of Xi’an Jiaotong University, Xi’an, Shaanxi, China; 3 Department of Cell Biology and Genetics, School of Basic Medical Sciences, Xi’an Jiaotong University Health Science Center, Xi’an, Shaanxi, China

**Keywords:** 2-hydroxy-3-methylbutyric acid, HPLC–UV, impaired semen quality, male infertility, nucleotide metabolism, seminal plasma, untargeted metabolomics

## Abstract

**Background:**

Impaired semen quality may involve concurrent abnormalities in sperm concentration, total sperm number, motility, morphology, and DNA integrity. The corresponding alterations in the seminal plasma metabolome remain incompletely characterized.

**Objectives:**

To characterize seminal plasma metabolic alterations in men with multiple concurrent semen abnormalities and identify candidate signals associated with semen quality parameters.

**Materials and methods:**

Untargeted LC–MS/MS metabolomics was performed in a discovery cohort comprising 10 men with impaired semen quality and 9 normozoospermic controls. Candidate metabolites were screened using nominal p value and fold-change thresholds, followed by pathway and correlation analyses, *post hoc* ROC analysis, and qualitative comparisons with public datasets. External-calibration-derived estimates assigned to cytidine and 2-hydroxy-3-methylbutyric acid (2-HMBA) were subsequently evaluated by HPLC–UV in an independent cohort of 18 men with impaired semen quality and 18 controls.

**Results:**

Eighty-nine putatively annotated metabolites met the nominal screening criteria, but no individual metabolite remained significant after Benjamini–Hochberg false discovery rate correction. Discovery-level pathway analyses highlighted nucleotide- and nucleoside-related processes as hypothesis-generating signals. Qualitative comparison with heterogeneous publicly available reproductive metabolomics datasets provided limited directional context but was not considered formal external validation. In the independent HPLC–UV cohort, external-calibration-derived estimates assigned to 2-HMBA were nominally lower in the group with impaired semen quality (p = 0.030), but the difference did not remain significant after correction across the two candidate comparisons (q = 0.060). Cytidine showed no between-group difference (p = 0.812; q = 0.812).

**Discussion and conclusion:**

The discovery-level nucleotide-related pathway signal and the independent observation concerning 2-HMBA represent distinct lines of evidence; the latter does not validate the former. Given the small sample sizes in both the discovery and independent HPLC–UV evaluation cohorts, the absence of FDR-significant metabolites, heterogeneity across the public datasets, and limitations of the HPLC–UV assay, these findings remain exploratory and hypothesis-generating. They do not constitute quantitative validation, establish a specific mechanism, or support clinical biomarker use. Larger multicenter cohorts and targeted quantitative metabolomic analyses using validated methods are needed.

## 1 Introduction

Impaired semen quality encompasses abnormalities in conventional semen characteristics, such as sperm concentration, motility, and morphology, and may coexist with impaired sperm DNA integrity. It is an important contributor to male infertility, with male factors estimated to be involved in approximately 30–50% of infertility cases worldwide (1). Recent systematic reviews and meta-analyses have reported lower total and progressive sperm motility among male partners of couples with recurrent pregnancy loss than among controls (2). Although these associations do not establish causality, they highlight the relevance of sperm motility and overall male gamete quality to reproductive outcomes beyond conception. However, reduced sperm motility frequently coexists with abnormalities in other semen characteristics.

Accordingly, because the affected participants in the present study exhibited abnormalities extending beyond motility, the study was framed around multidimensionally impaired semen quality rather than isolated asthenozoospermia. Although conventional semen analysis remains central to the clinical assessment of male fertility, it primarily describes phenotypic characteristics and provides limited insight into the molecular and metabolic disturbances underlying sperm dysfunction (3). Moreover, conventional semen parameters may not fully reflect the molecular and biochemical heterogeneity underlying impaired semen quality, supporting the investigation of complementary omics-based markers (4). A better understanding of the associated molecular and metabolic alterations is therefore needed to clarify the biological processes underlying male reproductive dysfunction.

Seminal plasma provides the extracellular biochemical environment surrounding ejaculated sperm and contains metabolites derived from multiple components of the male reproductive tract. It has therefore emerged as an informative matrix for investigating metabolic alterations associated with sperm dysfunction and impaired semen quality (5). Previous omics-based and experimental studies have implicated alterations in energy metabolism, oxidative stress, lipid remodeling, inflammatory signaling, and mitochondrial function in male infertility and abnormal semen phenotypes (6–9). However, reported findings have varied across studies, partly because of differences in biological matrix, phenotype definition, analytical platform, data preprocessing, and metabolite annotation. Consequently, the reproducibility of individual metabolite alterations and their relationships with clinically relevant semen characteristics remains uncertain.

In this exploratory study, we combined untargeted LC–MS/MS profiling of seminal plasma with pathway and metabolite–phenotype analyses to characterize metabolic alterations associated with multidimensionally impaired semen quality. We further examined the directional consistency of selected candidates in heterogeneous publicly available reproductive metabolomics datasets and performed an independent HPLC–UV evaluation of external-calibration-derived estimates assigned to selected candidates. The study was designed to generate and prioritize biologically plausible candidate signals for subsequent analytical and biological investigation rather than to establish clinical diagnostic utility.

## 2 Materials and methods

### 2.1 Study design and participants

**Figure 1:**
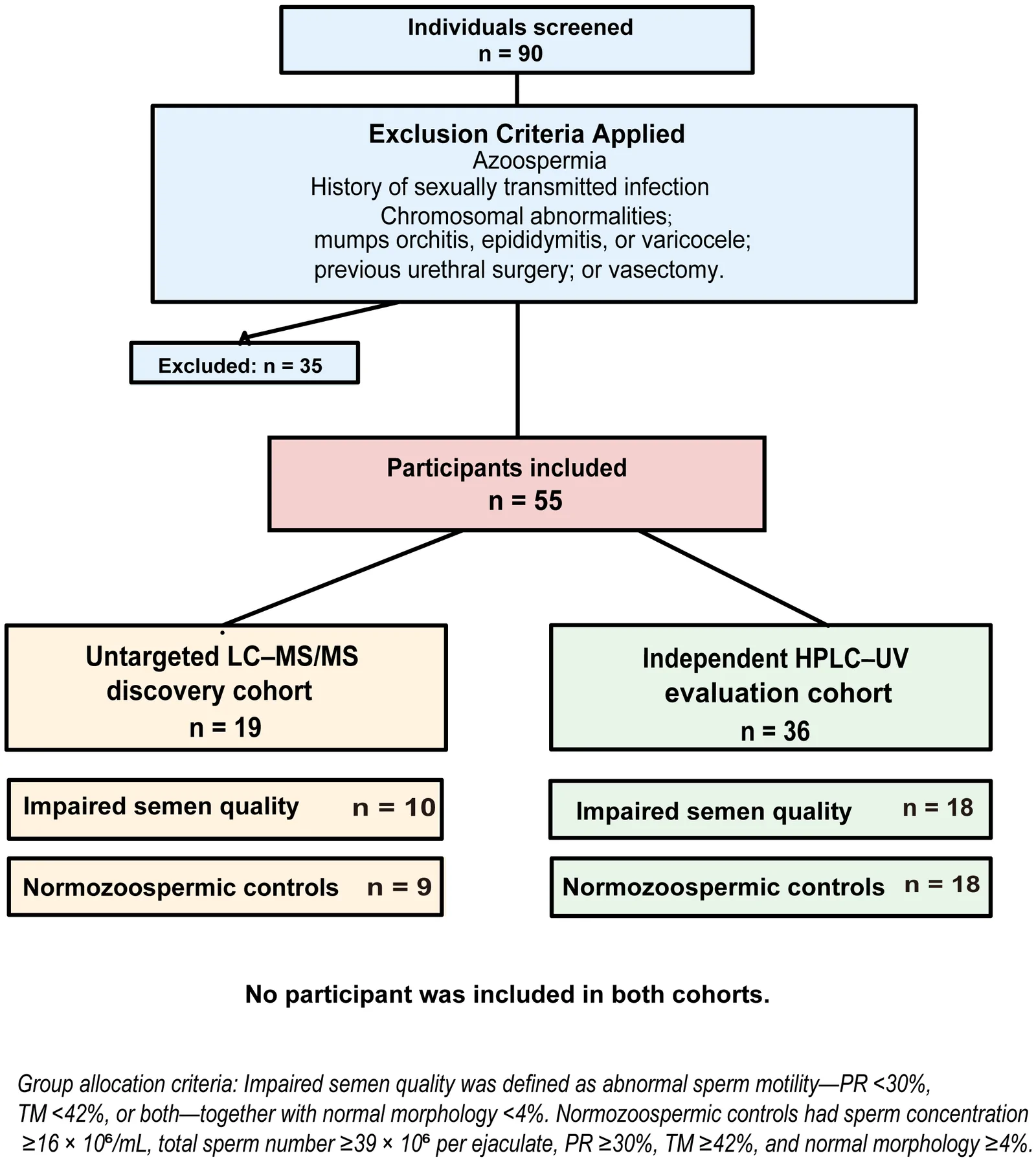
Study design and participant selection workflow. A total of 90 individuals were initially screened. After application of the predefined eligibility criteria, 55 participants were included across two independent cohorts. The untargeted discovery cohort comprised 10 men with impaired semen quality and 9 normozoospermic controls. The independent HPLC–UV evaluation cohort comprised 18 men with impaired semen quality and 18 normozoospermic controls. Group allocation was based on study-defined combinations of the lower reference values for semen parameters reported in the sixth edition of the WHO Laboratory Manual for the Examination and Processing of Human Semen (10). PR, progressive motility; TM, total motility.

This hospital-based case–control study was conducted at the First Affiliated Hospital of Xi’an Jiaotong University. Men undergoing semen analysis at the andrology laboratory between January and August 2025 were recruited. A total of 55 participants were included across two independent cohorts. The untargeted discovery cohort comprised 10 men with impaired semen quality and 9 normozoospermic controls, whereas the independent HPLC–UV evaluation cohort comprised 18 men with impaired semen quality and 18 normozoospermic controls. No participant was included in both cohorts. The participant enrollment and cohort allocation workflow is shown in **Figure 1**.

Participants were excluded if they had azoospermia, a history of sexually transmitted infection, chromosomal abnormalities, mumps orchitis, epididymitis, varicocele, previous urethral surgery, or vasectomy.

Participants were included in the impaired semen quality group if they had sperm motility below the WHO lower reference limits—progressive motility <30%, total motility <42%, or both—and concurrently exhibited abnormal sperm morphology, defined as normal morphology <4% (10). Because abnormal morphology was required in addition to reduced sperm motility, the affected cohort did not represent isolated asthenozoospermia.

Normozoospermic controls were required to have sperm concentration, total sperm number, progressive motility, total motility, and normal morphology at or above the corresponding WHO lower reference limits. Cohort allocation was completed before metabolomic analysis.

### 2.2 Ethical approval and informed consent

The study protocol was approved by the Institutional Review Board of the First Affiliated Hospital of Xi’an Jiaotong University (IRB No. XJTU1AF2025LSYY-650). Written informed consent was obtained from all participants prior to enrollment.

### 2.3 Semen collection and semen quality assessment

After 2–7 days of sexual abstinence, semen samples were collected by masturbation into sterile containers and allowed to liquefy at 37 °C for 30 minutes. Routine semen parameters were assessed according to the World Health Organization (WHO) Laboratory Manual (6th edition). Sperm concentration and motility were measured using a computer-assisted sperm analysis (CASA) system, and sperm morphology was evaluated according to WHO strict morphological criteria. For reference, the WHO lower reference limits for basic semen parameters were as follows: semen volume ≥ 1.4 mL, pH ≥ 7.2, sperm concentration ≥ 16 × 10^6^/mL, total sperm number (per ejaculation) ≥ 39 × 10^6^, progressive motility ≥ 30%, total motility ≥ 42%, and normal morphology ≥ 4%.

Total sperm number was calculated as semen volume × sperm concentration. Total progressive sperm count was calculated as semen volume × sperm concentration × progressive motility (fraction). Sperm DNA fragmentation index (DFI) and high DNA stainability (HDS) were determined using the sperm chromatin structure assay (SCSA) performed according to the manufacturer’s instructions. DFI and HDS (%) were recorded as additional semen-quality characteristics and were not used for cohort allocation.

### 2.4 Clinical information and potential confounders

Clinical information, including age, body mass index (BMI), and abstinence duration, was recorded. Information on smoking, alcohol consumption, dietary habits, medication use, metabolic syndrome-related variables, leukocytospermia, and inflammatory status was not systematically available and could not be included in adjusted analyses.

### 2.5 Seminal plasma preparation

After liquefaction and completion of routine semen analysis, samples were centrifuged at 3,000 × g for 10 min at 4 °C. The seminal plasma supernatant was carefully collected, aliquoted, and stored at −80 °C within 1 h after ejaculation. The interval between sample collection and metabolomic analysis did not exceed 8 months, and samples underwent only one freeze–thaw cycle before extraction.

### 2.6 Untargeted metabolomics analysis

#### 2.6.1 Sample preparation

Frozen seminal plasma samples stored at −80 °C were thawed on ice. An aliquot of 100 μL from each sample was transferred to a 1.5-mL microcentrifuge tube. Protein precipitation was performed by adding 400 μL of methanol: acetonitrile (2:1, v/v) containing internal standards, followed by vortexing for 1 minute. Samples were sonicated in an ice-water bath for 10 minutes and incubated at −40 °C overnight. After centrifugation at 12,000 × g at 4 °C for 20 minutes to further remove precipitated proteins and residual particulate matter, 150 μL of the supernatant was transferred to LC–MS vials.

A pooled quality-control (QC) sample was prepared by combining equal volumes of extract from all individual samples. QC samples and internal standards were periodically injected throughout the analytical sequence to monitor instrument stability and reproducibility.

#### 2.6.2 LC–MS/MS acquisition

LC–MS/MS experiments were conducted on a Waters ACQUITY UPLC I-Class Plus system coupled to a Thermo Q Exactive Plus mass spectrometer. A 5-μL aliquot was injected onto an ACQUITY UPLC HSS T3 column (100 mm × 2.1 mm, 1.8 μm) maintained at 45 °C. The mobile phase consisted of (A) water containing 0.1% formic acid and (B) acetonitrile. Chromatographic separation was performed at a flow rate of 0.35 mL/min using the following gradient: 5% B was maintained from 0 to 2.0 min; B was increased to 30% from 2.0 to 4.0 min, to 50% from 4.0 to 8.0 min, to 80% from 8.0 to 10.0 min, and to 100% from 10.0 to 14.0 min. The mobile phase was maintained at 100% B until 15.0 min, returned to 5% B at 15.1 min, and maintained at 5% B until 16.0 min for column re-equilibration.

Heated electrospray ionization (HESI) was used in both positive and negative ion modes. Data were acquired in data-dependent acquisition (DDA) mode with one full MS scan followed by MS/MS scans of the top 10 most intense precursor ions. Full MS resolution was set to 70,000 and MS/MS resolution to 17,500. Key parameters were: spray voltage 3,800 V (positive) and −3,200 V (negative); sheath gas flow rate 35 arbitrary units; capillary temperature 320 °C; mass range m/z 70–1,050; and normalized collision energy stepped at 10, 20, and 40 eV.

### 2.7 Independent HPLC–UV evaluation

#### 2.7.1 Candidate selection and reference standards

An independent HPLC–UV evaluation was conducted in the separate cohort described above to compare external-calibration-derived estimates assigned to two prioritized metabolites between groups. Candidate prioritization was qualitative and considered complementary exploratory evidence from discovery-stage group comparisons, pathway analyses, descriptive *post hoc* ROC analyses, IPA knowledge-base contextualization, and qualitative cross-dataset comparisons. No formal composite score or prespecified weighting scheme was applied.

Cytidine was selected as a pathway-associated candidate representing the exploratory nucleotide- and nucleoside-related signal, whereas 2-HMBA was selected as a separate individual-metabolite candidate on the basis of its discovery-stage and cross-dataset evidence together with IPA-derived biological context. The subsequent HPLC–UV analysis was designed as an exploratory orthogonal evaluation rather than analyte-specific quantitative validation.

Authentic cytidine (CAS No. 65-46-3; Catalog No. ZC-51672; purity, 95.41%) and 2-HMBA (CAS No. 4026-18-0; Catalog No. ZC-54943; purity, 95.22%) reference standards were purchased from Shanghai ZZBIO Co., Ltd. (Shanghai, China). Stock and working standard solutions were prepared in methanol.

#### 2.7.2 Sample preparation

Frozen seminal plasma samples were thawed on ice immediately before analysis. An aliquot of 50 μL of seminal plasma was mixed with 150 μL of methanol, vortexed for 1 min, and centrifuged at 8,000 rpm for 10 min at 4 °C. The resulting supernatant was collected and passed through a 0.22-μm membrane filter before HPLC–UV analysis. The nominal total extraction volume was 200 μL. All biological samples were processed using the same extraction procedure.

#### 2.7.3 HPLC–UV conditions

HPLC–UV analysis was performed using an Agilent 1260 Infinity II liquid chromatography system equipped with a variable wavelength detector. Chromatographic separation was achieved using a Welch Ultimate OAA column (300 × 4.6 mm) without active column-temperature control and at ambient laboratory temperature. The mobile phase consisted of water containing 0.1% formic acid and acetonitrile at a fixed ratio of 60:40 (v/v) under isocratic conditions. The flow rate was 0.5 mL/min, the injection volume was 10 μL, and UV detection was performed at 205 nm. The total chromatographic run time was 10 min.

#### 2.7.4 External calibration and concentration estimation

Five-point external calibration curves were established over extract-level concentration ranges of 20–100 μg/mL for cytidine and 200–1,000 μg/mL for 2-HMBA. Calibration curves were generated by plotting the integrated peak-area response against the nominal standard concentration using unweighted linear regression constrained through the origin. Chromatographic peak integration, calibration-curve fitting, and response-derived estimation were performed using the Agilent MassHunter quantitative batch-processing environment, version 10.2. Detailed calibration equations, coefficients of determination, and point-level back-calculated results are provided in **Supplementary Table 2** and **Supplementary Figure 2**.

For each biological sample, the integrated peak-area response was converted using the corresponding external calibration equation to an estimated concentration in the processed methanolic extract. Because 50 μL of seminal plasma was extracted to a nominal total volume of 200 μL, the calculated extract value was multiplied by four and expressed on a nominal seminal-plasma concentration scale (μg/mL). These values are referred to throughout as external-calibration-derived estimates rather than validated quantitative concentrations.

Values corresponding to responses below the lowest calibration level were obtained by downward extrapolation of the fitted regression equation. Such values were retained only as exploratory, response-derived estimates for relative between-group comparison and were not interpreted as measurements within a validated quantitative range.

#### 2.7.5 Peak assignment and analytical quality assessment

Calibration standards, a solvent blank, and the biological samples were analyzed within the same analytical sequence. Candidate peaks in biological samples were assigned using analyte-specific retention-time regions configured in the MassHunter quantitative method, with reference to the chromatographic behavior of the authentic standards and the concentration-dependent responses observed across the calibration series. Retention-time variation in the biological matrix was evaluated within the predefined analyte-specific integration regions. Peak shape, baseline placement, and integrated peak-area response were manually reviewed before data export.

No reportable integrated analyte peak was detected in the solvent blank within the predefined retention-time regions used for cytidine and 2-HMBA assessment. Representative mixed-standard and solvent-blank chromatograms are provided in **Supplementary Figure 2**, and representative chromatographic traces from one normozoospermic control and one participant with impaired semen quality are shown in **Supplementary Figure 3**. Signal estimation was performed without an internal standard, and no pooled targeted quality-control sample was included in the HPLC–UV analytical sequence.

Formal peak-purity assessment was not performed. The signals in biological samples fell within the predefined analyte-specific retention-time regions and were assigned with reference to authentic standards, although modest retention-time variation was observed in the biological matrix. Because detection was conducted at a single UV wavelength and no sample-level MS/MS or standard-addition confirmation was available, contributions from co-eluting matrix components could not be excluded.

### 2.8 Untargeted data processing and metabolite annotation

Raw data files were processed using XCMS (version 4.5.1) for baseline filtering, peak detection, peak integration, and retention-time alignment (11, 12). Peak detection and retention-time correction were performed using the centWave algorithm with parameters optimized for high-resolution LC–MS data.

Metabolite annotation was performed by matching detected features against the Human Metabolome Database (HMDB) (13), LIPID MAPS (version 2.3) (14), METLIN (15), and the LuMet-Animal 3.0 database. An internally defined composite scoring system incorporating accurate precursor-mass matching, MS/MS fragmentation matching, isotope-distribution matching, and retention-time matching was used to assess annotation confidence. Each component contributed up to 20 points, yielding a maximum composite score of 80. Putative annotations with a composite score below 36 were excluded from downstream analysis. Because the annotations were not confirmed by direct comparison with authentic reference standards, the retained compounds were considered putatively annotated metabolites rather than definitively identified metabolites. The final merged matrix contained 2,612 entries with unique metabolite names and InChIKeys, and no duplicate InChIKeys were present across positive- and negative-ionization modes.

After annotation filtering, data acquired in the positive- and negative-ionization modes were merged into a single data matrix. Features with a relative standard deviation greater than 30% in the pooled QC samples were excluded. Features with zero or missing values in more than 50% of samples within either study group were also removed. For the remaining features, zero or missing values were replaced with one-half of the minimum non-zero ion intensity observed across the complete retained dataset. The resulting intensity matrix was subsequently log2-transformed and used for downstream statistical analyses.

### 2.9 Statistical and bioinformatic analyses

Unless otherwise specified, statistical analyses were performed using R software (version 4.5.2), and all statistical tests were two-sided. A nominal p value <0.05 was used for exploratory screening unless otherwise specified. Benjamini–Hochberg false discovery rate correction was applied separately within the prespecified analysis families, including the 2,612 putatively annotated metabolites, the 3,916 metabolite–metabolite correlation tests, the metabolite–semen-quality-parameter correlation tests, the KEGG and Reactome pathway analyses considered separately, and the two candidate-metabolite comparisons in the independent HPLC–UV cohort. Correlations involving age and BMI were treated separately as descriptive analyses and were not included in the metabolite–semen-quality-parameter multiple-testing family. Adjusted q values were calculated within each family. IPA canonical-pathway results were interpreted descriptively using nominal IPA enrichment p values, as described in Section 2.11.

#### 2.9.1 Baseline demographic and semen-characteristic analyses

Baseline variables were summarized and compared separately within the untargeted discovery cohort and the independent HPLC–UV evaluation cohort. Normally distributed variables are presented as mean ± SD and were compared using Student’s t-test when variances were homogeneous or Welch’s t-test otherwise. Normally distributed variables were summarized as mean ± SD and compared using Student’s t-test when variances were homogeneous or Welch’s t-test otherwise. Non-normally distributed variables were summarized as median (Q1–Q3) and compared using the Mann–Whitney U test.

#### 2.9.2 Multivariate analyses

Features acquired in both positive and negative ionization modes were analyzed using principal component analysis (PCA) and orthogonal partial least squares–discriminant analysis (OPLS–DA) in MetaboAnalyst 6.0 (16). PCA was used for unsupervised visualization of global metabolic variation and potential outliers, whereas OPLS–DA was used as an exploratory supervised approach to visualize group-associated metabolic patterns. Model fit was summarized using cumulative R²Y [R²Y(cum)], and predictive performance was assessed using cumulative Q² [Q²(cum)] estimated by seven-fold cross-validation. Model robustness and the potential for overfitting were further examined using response permutation testing with 200 permutations of the group labels. The permutation distributions and the regression-line intercepts at zero label similarity were used as supplementary model diagnostics.

#### 2.9.3 Exploratory candidate metabolite screening

VIP scores derived from the exploratory OPLS–DA model were retained as supportive descriptive information but were not included in the eligibility criteria for candidate-metabolite screening. Between-group p values were calculated separately for each of the 2,612 putatively annotated metabolites using one-way analysis of variance (ANOVA) applied to the imputed and log2-transformed intensity data, with study group included as a two-level factor (impaired semen quality versus normozoospermic control).

For each metabolite, log2 fold change was calculated as the difference between the mean log2-transformed abundance in the impaired semen quality group and that in the normozoospermic control group. Conventional fold change was obtained by back-transformation as FC = 2^(log2FC).

Exploratory candidate metabolites were defined using a conventional fold-change threshold of ≥1.5 or ≤0.67 together with a nominal p value <0.05. Benjamini–Hochberg correction was applied jointly across the p values from all 2,612 putatively annotated metabolites, and q < 0.05 was considered statistically significant after multiple-testing correction. Metabolites meeting the fold-change and nominal p-value criteria were retained as exploratory candidates for downstream hypothesis-generating analyses, including pathway enrichment, correlation and network analyses, *post hoc* ROC analyses, qualitative cross-dataset comparison, and candidate prioritization for independent HPLC–UV evaluation.

#### 2.9.4 Pathway enrichment analysis

Pathway-enrichment analyses were performed using the exploratory candidate metabolites meeting the nominal fold-change and p-value screening criteria. For KEGG over-representation analysis, duplicate annotations mapping to the same KEGG compound ID were collapsed to a single entry. The background universe comprised 447 unique KEGG compound IDs mapped from all 2,612 putatively annotated metabolites retained after quality-control filtering. The foreground set comprised the unique KEGG compound IDs mapped from the 89 exploratory candidate metabolites. KEGG enrichment analysis was conducted using MetaboAnalyst 6.0, and Reactome enrichment analysis was performed using the ReactomePA package in R (17–21). KEGG enrichment was assessed using the hypergeometric test. Benjamini–Hochberg correction was applied separately across all pathways tested within each framework, with KEGG and Reactome treated as distinct multiple-testing families. Both nominal p values and adjusted q values were retained, and findings with q < 0.10 were interpreted as suggestive pathway-level signals rather than confirmatory evidence.

#### 2.9.5 Correlation and network analyses

Pairwise Spearman correlations were calculated among all 89 exploratory candidate metabolites, resulting in 3,916 unique metabolite pairs. The corresponding p values were adjusted jointly across all 3,916 tests using the Benjamini–Hochberg procedure, and correlations meeting q ≤ 0.05 were considered FDR significant. Metabolites participating in at least one FDR-significant correlation were retained for network analysis. For visual legibility, **Supplementary Figure 1B** displays the 250 FDR-significant correlations with the largest absolute Spearman correlation coefficients; this display restriction was not used for statistical inference.

**Figure 2:**
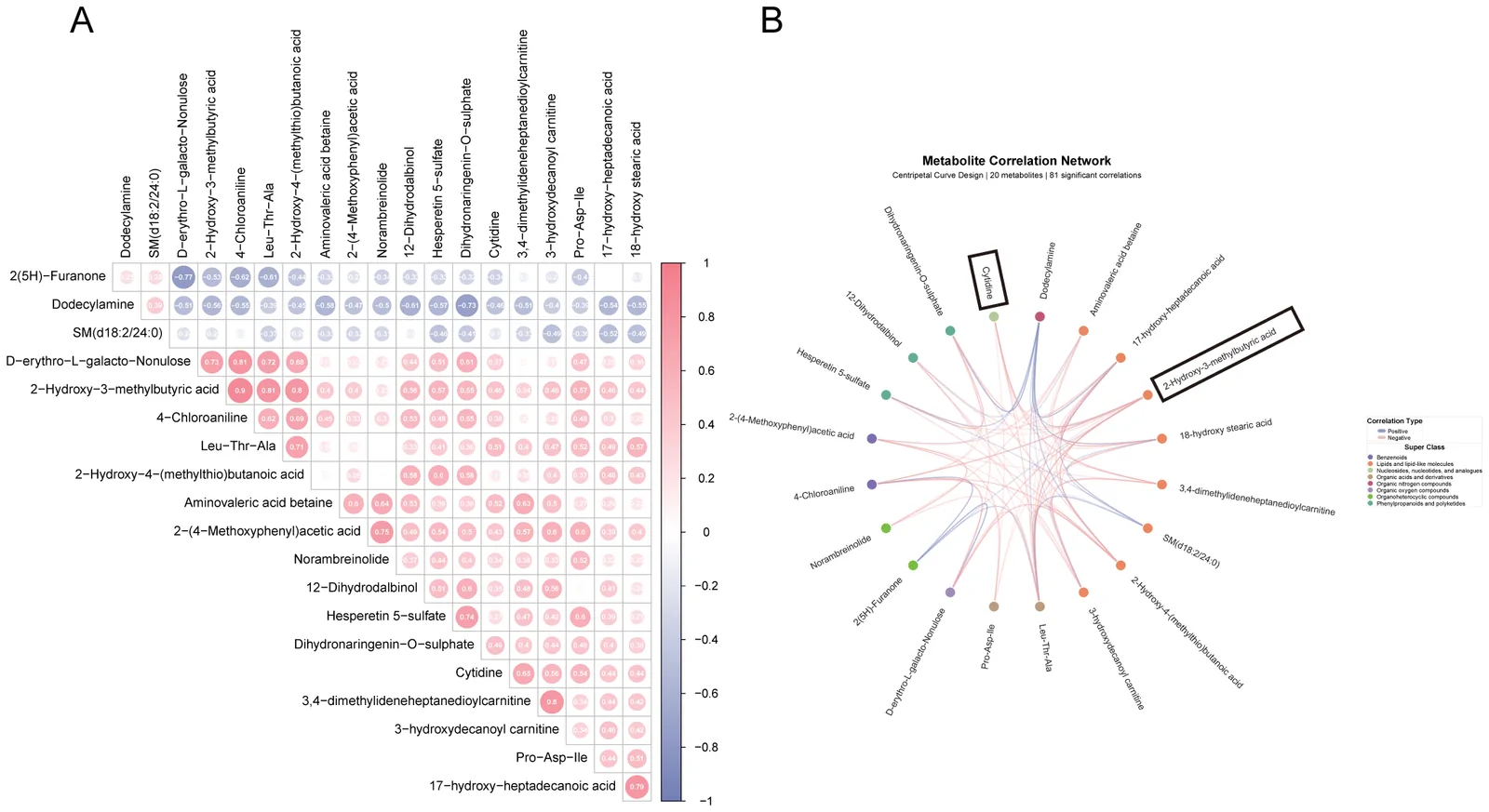
Focused visualization of metabolite correlations derived from the complete 89-metabolite analysis. **(A)** Pairwise Spearman correlation coefficients among the 20 displayed metabolites. The coefficients were extracted from the complete 89-metabolite correlation matrix. **(B)** Correlation network retaining only associations between the displayed metabolites that met q ≤ 0.05 after Benjamini–Hochberg correction across all 3,916 unique metabolite pairs. Node colors indicate metabolite superclass, and edge colors indicate correlation direction. Cytidine and 2-HMBA were boxed to denote their subsequent exploratory HPLC–UV evaluation. The 20 metabolites represent a focused visualization subset rather than an independently validated metabolite panel.

For clarity, a focused subset of 20 metabolites was selected for visualization in the main manuscript based on absolute correlation strength, connectivity within the full FDR-corrected network, and nominal between-group p values. No prespecified weighting scheme or formal composite score was used. The correlation coefficients displayed in **Figure 2A** and the q values used to construct **Figure 2B** were extracted from the complete 89-metabolite analysis. **Figure 2B** retained only correlations between the 20 displayed metabolites that met q ≤ 0.05 after Benjamini–Hochberg correction across all 3,916 tests; FDR correction was not recalculated within the 20-metabolite subset.

Spearman correlations were also calculated between the two prioritized candidate metabolites and semen-quality parameters. The p values from all metabolite–semen-quality-parameter correlations were adjusted jointly as a single multiple-testing family using the Benjamini–Hochberg procedure, with q < 0.05 considered statistically significant. Correlations with age and BMI were examined descriptively using nominal p values and were not included in this FDR-correction family.

#### 2.9.6 Exploratory classification analyses

*Post hoc* univariate ROC analyses were performed for cytidine and 2-HMBA in the untargeted discovery cohort using the pROC package (version 1.18.0). AUCs and 95% confidence intervals were estimated by bootstrap resampling. Because these analyses were *post hoc* and used the same discovery-cohort data that informed candidate prioritization, the resulting AUCs were interpreted as descriptive measures of within-cohort group separation rather than as independent estimates of diagnostic performance.

#### 2.9.7 Statistical analysis of the independent HPLC–UV evaluation

Between-group comparisons of the external-calibration-derived estimates assigned to cytidine and 2-HMBA were performed using the Mann–Whitney U test. Benjamini–Hochberg correction was applied jointly across the two candidate comparisons, and both nominal p values and FDR-adjusted q values were reported.

An exploratory *post hoc* ROC analysis was performed for the estimates assigned to 2-HMBA using the pROC package. The AUC and its 95% confidence interval were estimated by bootstrap resampling. No prespecified classification threshold was evaluated, and the ROC analysis was not interpreted as evidence of diagnostic performance or clinical utility.

### 2.10 Cross-dataset comparative analysis

To provide qualitative cross-dataset directional context for cytidine and 2-HMBA, two publicly available reproductive metabolomics datasets were examined: MTBLS5289 from MetaboLights (22) and ST003266 from Metabolomics Workbench (23)(Project ID PR002028; Project DOI: 10.21228/M8GC01). Processed metabolite-abundance matrices provided by the respective repositories were used.

When a candidate metabolite was available, its direction of change was compared qualitatively with that observed in the present discovery cohort. Dataset-internal group comparisons and *post hoc* ROC curves were also generated descriptively for visualization. Because the external studies differed from the present study in biological matrix, cohort phenotype, analytical platform, preprocessing procedures, and metabolite-annotation confidence, they were not treated as formal external-validation cohorts or used for model replication. Detailed dataset characteristics and major limitations in comparability are summarized in **Supplementary Table 1**.

### 2.11 Ingenuity pathway analysis

Ingenuity Pathway Analysis (IPA; QIAGEN, Redwood City, CA, USA) was used for canonical-pathway analysis and knowledge-base-derived network contextualization of the exploratory candidate-metabolite list (24). Candidate-metabolite identifiers and corresponding abundance changes were uploaded to IPA and mapped to entities in the QIAGEN Knowledge Base.

Canonical pathways were ranked according to the nominal IPA enrichment p value, expressed as −log10(p). These nominal IPA p values were interpreted descriptively and were not treated as FDR-confirmed pathway findings. Network analysis incorporated direct and indirect curated relationships supported by experimentally observed evidence, with endogenous chemicals included. IPA network outputs and curated relationships relevant to the prioritized metabolites were summarized in an integrated knowledge-base-derived interaction map for exploratory biological contextualization.

## 3 Results

### 3.1 Baseline demographic and semen characteristics of the study population

Of the 90 individuals initially screened, 55 participants were included, comprising 19 participants in the untargeted discovery cohort (10 men with impaired semen quality and 9 controls) and 36 participants in the independent HPLC–UV evaluation cohort (18 and 18, respectively) (**Figure 1**). Baseline characteristics were evaluated separately within each cohort (**Table 1**). Age, BMI, abstinence duration, and semen volume did not differ significantly between groups in either cohort, whereas multiple conventional semen quality parameters showed the expected case–control differences.

**Table 1:** Baseline demographic, preanalytical, and semen characteristics of the untargeted discovery cohort and the independent HPLC–UV evaluation cohort.

Variable	Untargeted discovery cohort			Independent HPLC–UV evaluation cohort		
	Control (n = 9)	Impaired semen quality (n = 10)	P value	Control (n = 18)	Impaired semen quality (n = 18)	P value
Demographic and preanalytical characteristics						
Age (years)	30.00 (29.75, 34.00)	32.00 (30.00, 35.25)	0.318	32.00 (29.25, 34.00)	31.50 (30.25, 34.00)	0.869
BMI (kg/m²)	25.64 ± 2.26	25.18 ± 1.69	0.456	25.58 ± 2.20	24.76 ± 1.27	0.181
Abstinence duration (days)	4.00 (3.00, 6.00)	4.00 (3.00, 5.00)	0.676	4.00 (3.00, 5.00)	4.00 (3.00, 5.00)	0.758
Semen volume (mL)	3.20 (2.95,4.1)	3.20 (2.63,3.98)	0.253	3.35 (3.10, 4.08)	3.05 (2.45, 3.88)	0.167
Semen quality characteristics						
Sperm concentration (×10^6^/mL)	123.10 ± 57.89	23.78 ± 18.90	<0.001	130.19 ± 39.49	17.03 ± 8.91	<0.001
Total sperm number (×10^6^ per ejaculate)	415.12 ± 204.77	77.49 ± 70.10	<0.001	470.96 ± 170.19	50.36 ± 21.01	<0.001
Total progressive sperm count (×10^6^ per ejaculate)	303.21 ± 164.10	19.22 ± 16.13	<0.001	338.01 ± 131.77	12.21 ± 7.49	<0.001
Progressive motility (%)	80.20 ± 9.30	22.40 ± 15.70	<0.001	71.51 ± 9.84	25.88 ± 13.55	<0.001
Total motility (%)	85.74 ± 7.67	35.38 ± 14.07	<0.001	84.54 ± 7.75	33.99 ± 13.32	<0.001
Normal morphology (%)	4.50 (4.20, 4.80)	2.10 (2.00, 2.50)	<0.001	4.70 (4.33, 4.80)	2.50 (2.00, 2.90)	<0.001
Sperm DNA fragmentation index (%)	12.90 (9.20, 14.70)	34.90 (19.60, 54.90)	<0.001	12.26 (9.56, 14.50)	35.33 (22.21, 51.20)	<0.001

Data are presented as mean ± standard deviation or median (first quartile, third quartile), as appropriate. Between-group comparisons were performed separately within each cohort using Student’s t-test, Welch’s t-test, or the Mann–Whitney U test, as appropriate. P values are descriptive and were not adjusted for multiple comparisons. BMI, body mass index.

Consistent with the cohort-allocation criteria, men in the impaired semen quality group had lower progressive motility, total motility, and normal morphology than controls. They also had lower sperm concentration, total sperm number, and total progressive sperm count, as well as a higher sperm DNA fragmentation index, indicating that the affected group had abnormalities across multiple semen parameters. Detailed values and between-group comparisons are provided in **Table 1**.

### 3.2 Exploratory metabolomic profiling of seminal plasma

**Figure 3:**
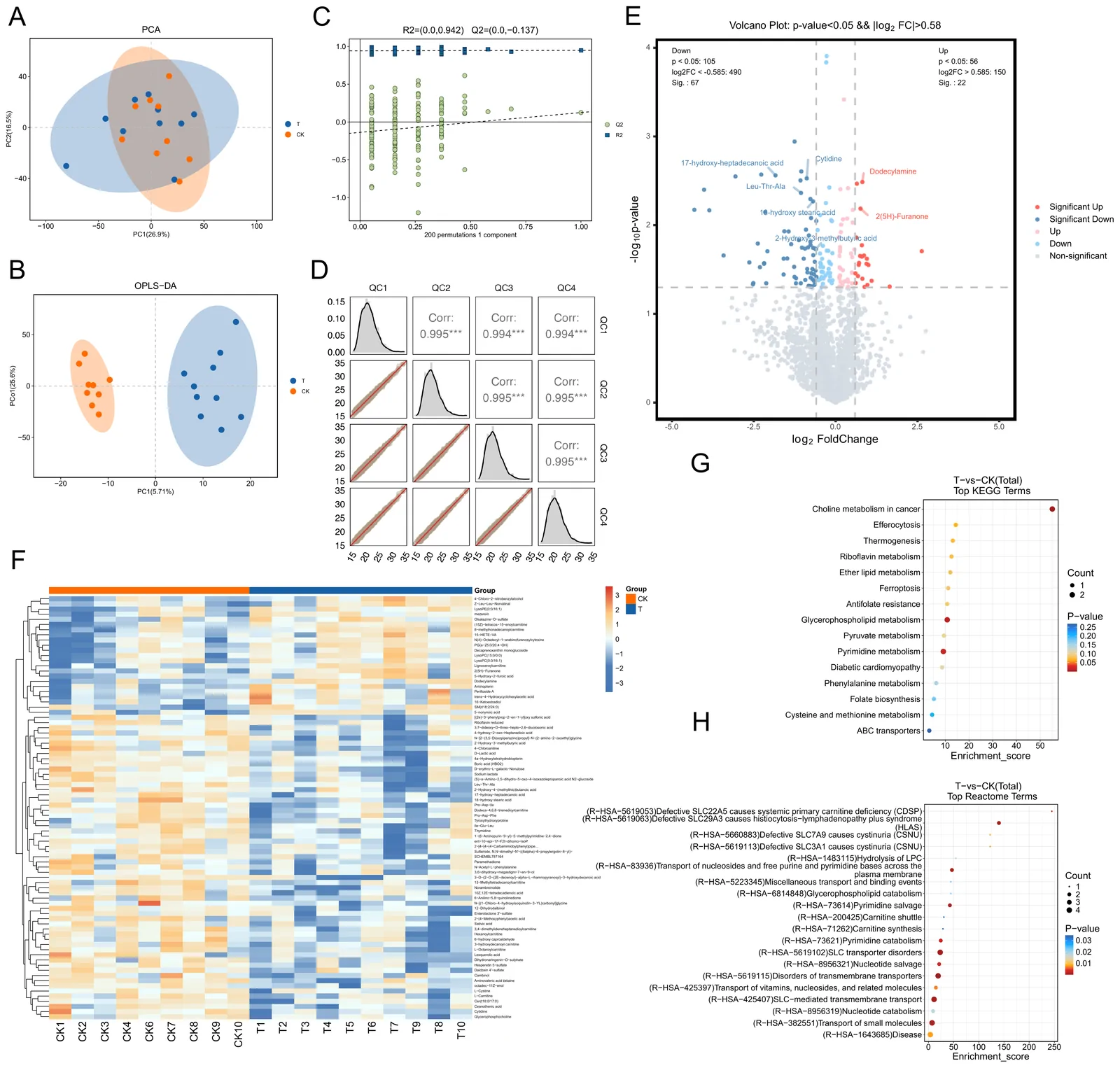
Exploratory untargeted metabolomic profiling of seminal plasma in men with impaired semen quality. **(A)** Principal component analysis (PCA) score plot showing partial separation between impaired semen quality and control samples. **(B)** Exploratory OPLS–DA score plot. The original model showed R²Y(cum) = 0.948 and a seven-fold cross-validated Q²(cum) = 0.125, indicating high in-sample fit but limited predictive performance. **(C)** Response permutation testing of the OPLS–DA model based on 200 permutations of the group labels. The regression-line intercepts at zero label similarity were 0.942 for R² and −0.137 for Q². These values represent permutation-regression intercepts rather than the original model’s R²Y(cum) and Q²(cum). The permutation results did not provide evidence of a robust predictive model. **(D)** Quality control (QC) correlation plot demonstrating the reproducibility and stability of the metabolomics data. **(E)** The volcano plot displays 89 metabolites meeting the prespecified fold-change and nominal p-value screening criteria. Benjamini–Hochberg correction was performed across all 2,612 metabolites included in the between-group analysis, and no individual metabolite remained significant at FDR-adjusted q < 0.05. **(F)** Heatmap of exploratory candidate metabolites meeting the nominal screening criteria. Rows represent metabolites and columns represent samples; red and blue indicate higher and lower relative abundance, respectively. **(G)** KEGG pathway-enrichment bubble plot. The x-axis shows the enrichment score, bubble size indicates the number of mapped metabolites, and color represents the nominal pathway p value. Benjamini–Hochberg-adjusted q values were calculated across all KEGG pathways and are reported in the Results. **(H)** Reactome pathway-enrichment bubble plot. The x-axis shows the enrichment score, bubble size indicates the number of mapped metabolites, and color represents the nominal pathway p value. Benjamini–Hochberg-adjusted q values were calculated across all Reactome pathways and are reported in the Results. CK, normozoospermic controls; T, men with impaired semen quality.

Untargeted metabolomic profiling was performed to evaluate global metabolic differences between men with impaired semen quality and normozoospermic controls. Principal component analysis (PCA) showed partial separation between the two groups, suggesting differences in seminal plasma metabolic composition (**Figure 3A**).

OPLS–DA produced apparent separation between the groups and showed a high goodness-of-fit value (R²Y(cum) = 0.948); however, the seven-fold cross-validated Q²(cum) value was only 0.125, indicating limited predictive performance (**Figure 3B**). Response permutation testing with 200 permutations yielded regression-line intercepts of 0.942 for R² and −0.137 for Q² at zero label similarity (**Figure 3C**). The high R² intercept, low cross-validated Q², and small discovery cohort did not support the robustness or generalizability of the supervised model. Accordingly, the OPLS–DA model and its derived VIP scores were interpreted only as descriptive exploratory outputs, and VIP was not used as an eligibility criterion for defining the 89 exploratory candidate metabolites. Pooled quality-control samples clustered tightly, with correlation coefficients exceeding 0.99 (**Figure 3D**), supporting the analytical stability of the platform.

### 3.3 Exploratory candidate metabolites associated with impaired semen quality

A total of 2,612 unique putatively annotated metabolite entries were included in the between-group analysis. Of these, 89 met the exploratory screening criteria of a fold change ≥1.5 or ≤0.67 together with a nominal p value <0.05, including 22 metabolites with higher abundance and 67 with lower abundance in the impaired semen quality group (**Figure 3E**). Benjamini–Hochberg correction was applied jointly to the p values from all 2,612 metabolite-level between-group tests, and no individual metabolite remained significant at q < 0.05. The 89 metabolites were therefore retained as exploratory candidates for downstream hypothesis-generating analyses. The exploratory candidate set mainly comprised organic acids and derivatives, lipids, and nucleotide- or nucleoside-related metabolites. Hierarchical clustering of the 89 candidates showed group-associated abundance patterns, with an overall tendency toward lower abundance in the impaired semen quality group (**Figure 3F**).

### 3.4 Exploratory pathway enrichment highlights nucleotide-related processes

Of the 89 exploratory candidate metabolites, 11 were mapped to KEGG pathways. Pyrimidine metabolism was identified as a suggestive pathway-level signal and included cytidine and thymidine (nominal p = 0.0189; FDR-adjusted q = 0.0945) (**Figure 3G**).

Reactome analysis mapped five exploratory candidates and identified several overlapping nucleotide- and nucleoside-related terms that met the pathway-level FDR threshold, including transport of nucleosides and free purine and pyrimidine bases (q = 0.0040), pyrimidine salvage (q = 0.0041), pyrimidine catabolism (q = 0.0110), nucleotide salvage (q = 0.0129), and nucleotide catabolism (q = 0.0399) (**Figure 3H**). These overlapping Reactome terms were primarily driven by the same two mapped nucleosides, cytidine and thymidine, and were therefore interpreted collectively as a single exploratory nucleotide-related signal rather than as independent pathway-level findings.

Together, the KEGG and Reactome analyses suggested a possible nucleotide- and nucleoside-related signal in the discovery dataset but did not demonstrate altered nucleotide metabolism.

### 3.5 IPA-based pathway and network contextualization

**Figure 4:**
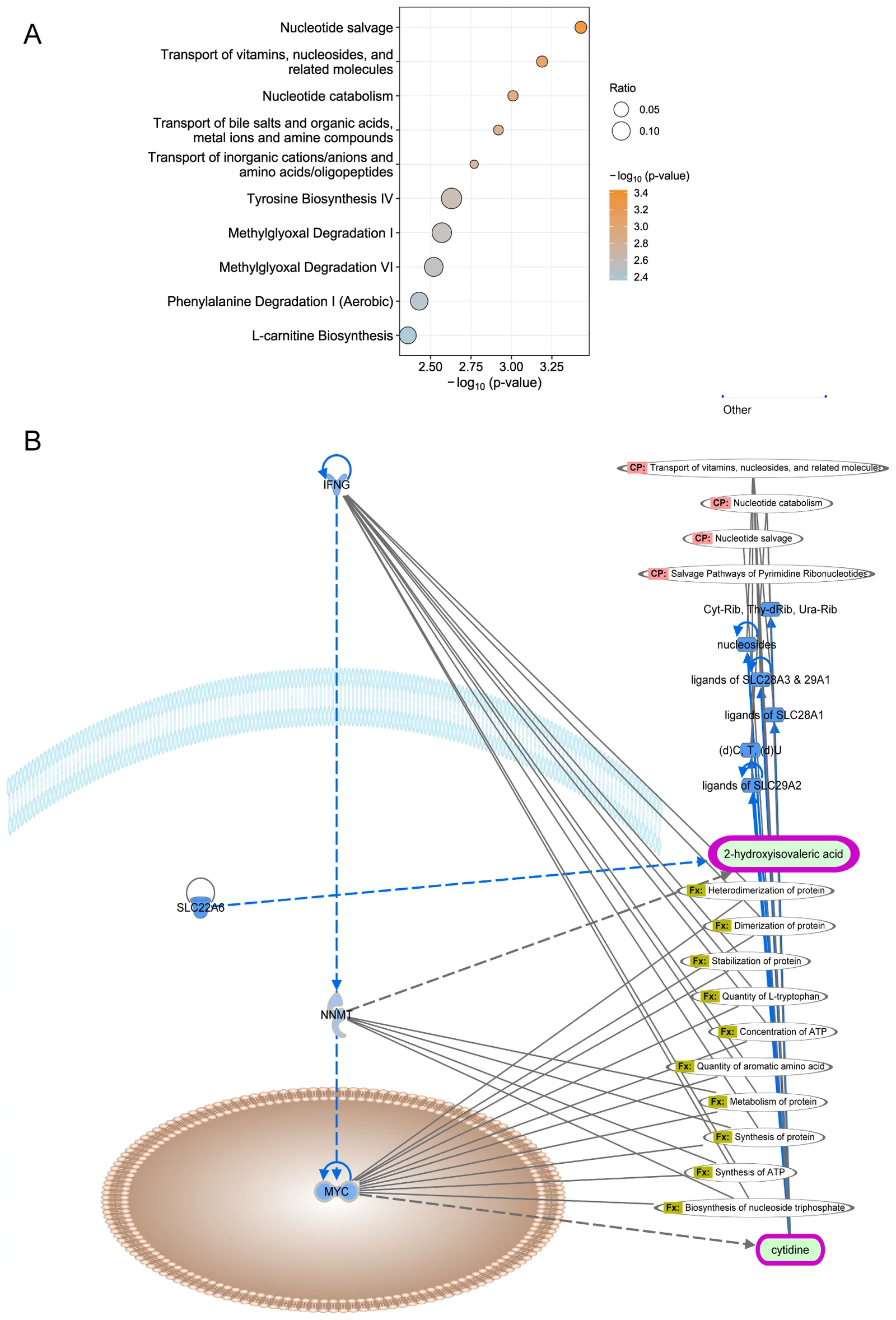
IPA-based pathway and network contextualization. **(A)** Author-generated bubble plot of the top-ranked IPA canonical pathways based on IPA-exported results. Pathways are ranked by −log10 of the nominal P value, and bubble size represents the pathway ratio. **(B)** IPA-generated interaction map exported directly without modification to the network content, contextualizing cytidine and 2-HMBA, which is shown in IPA as 2-hydroxyisovaleric acid. Relationships and functional or pathway nodes reflect curated IPA Knowledge Base annotations, not measurements made in this study. The map does not indicate that the metabolites belong to the same canonical pathway or form an experimentally validated regulatory network, and the placement of 2-HMBA does not imply involvement in nucleotide-related pathways.

Of the 89 exploratory candidate metabolites submitted to IPA, 31 were recognized as analysis-ready molecules. Canonical-pathway analysis, ranked according to nominal IPA enrichment p values, showed that the three highest-ranked terms were nucleotide salvage (−log10[p] = 3.43), transport of vitamins, nucleosides, and related molecules (−log10[p] = 3.19), and nucleotide catabolism (−log10[p] = 3.01) (**Figure 4A**). These overlapping nucleotide-related terms were supported primarily by cytidine and thymidine and were therefore interpreted collectively as an exploratory nucleotide/nucleoside-related signal rather than as independent pathway-level findings. Other top-ranked terms involved molecular transport, amino-acid-related metabolism, methylglyoxal degradation, and L-carnitine biosynthesis.

An integrated IPA-based interaction map was used to provide knowledge-base-derived context for the prioritized metabolites (**Figure 4B**). Within this visualization, cytidine was associated with nucleotide salvage, nucleotide catabolism, and nucleoside-transport-related annotations. In contrast, 2-HMBA was recognized in the IPA knowledge base as 2-hydroxyisovaleric acid and was contextualized through curated relationships involving SLC22A6 and NNMT. 2-HMBA was not included among the molecules supporting the IPA canonical-pathway results and was therefore not interpreted as evidence for the nucleotide-related pathway signal. The integrated map summarized curated knowledge-base connectivity and was not interpreted as evidence of shared canonical-pathway membership or experimentally demonstrated molecular regulation.

### 3.6 Metabolite co-variation patterns in the discovery cohort

Pairwise Spearman correlation analysis of the 89 exploratory candidate metabolites generated 3,916 unique metabolite pairs. Benjamini–Hochberg correction was applied jointly across all 3,916 pairwise tests. A total of 302 correlations involving 75 metabolites remained significant at q ≤ 0.05, with each of these 75 metabolites participating in at least one FDR-significant correlation. The complete 89-metabolite correlation matrix is presented in **Supplementary Figure 1A**. For visual clarity, **Supplementary Figure 1B** displays the 250 FDR-significant correlations with the largest absolute Spearman correlation coefficients rather than all 302 significant correlations.

For a more legible presentation in the main manuscript, **Figure 2** displays a focused subset of 20 metabolites. The Spearman correlation coefficients shown in **Figure 2A** were extracted from the complete 89-metabolite correlation matrix. Among the 190 possible unique correlations between the 20 displayed metabolites, 81 met q ≤ 0.05 based on the Benjamini–Hochberg correction across all 3,916 correlations in the complete analysis and were retained as network edges in **Figure 2B**. These network patterns describe statistical co-variation within the discovery cohort and do not establish direct biochemical interactions or causal regulation.

### 3.7 Qualitative cross-dataset comparison of selected candidates

Cytidine and 2-HMBA were prioritized for HPLC–UV evaluation using the qualitative, *post hoc* prioritization framework described in Section 2.7.1. Cytidine was retained as a representative of the discovery-level nucleotide- and nucleoside-related pathway signal, whereas 2-HMBA was prioritized as an individual candidate on the basis of its discovery-stage direction and magnitude, descriptive *post hoc* ROC performance, and directional agreement with MTBLS5289.

The direction of change for selected candidates was examined in two publicly available reproductive metabolomics datasets, MTBLS5289 and ST003266. Because these datasets differed from the present study in biological matrix, phenotype definition, analytical platform, preprocessing, and annotation confidence, they were used only for qualitative directional comparison and were not treated as formal external validation cohorts.

**Figure 5:**
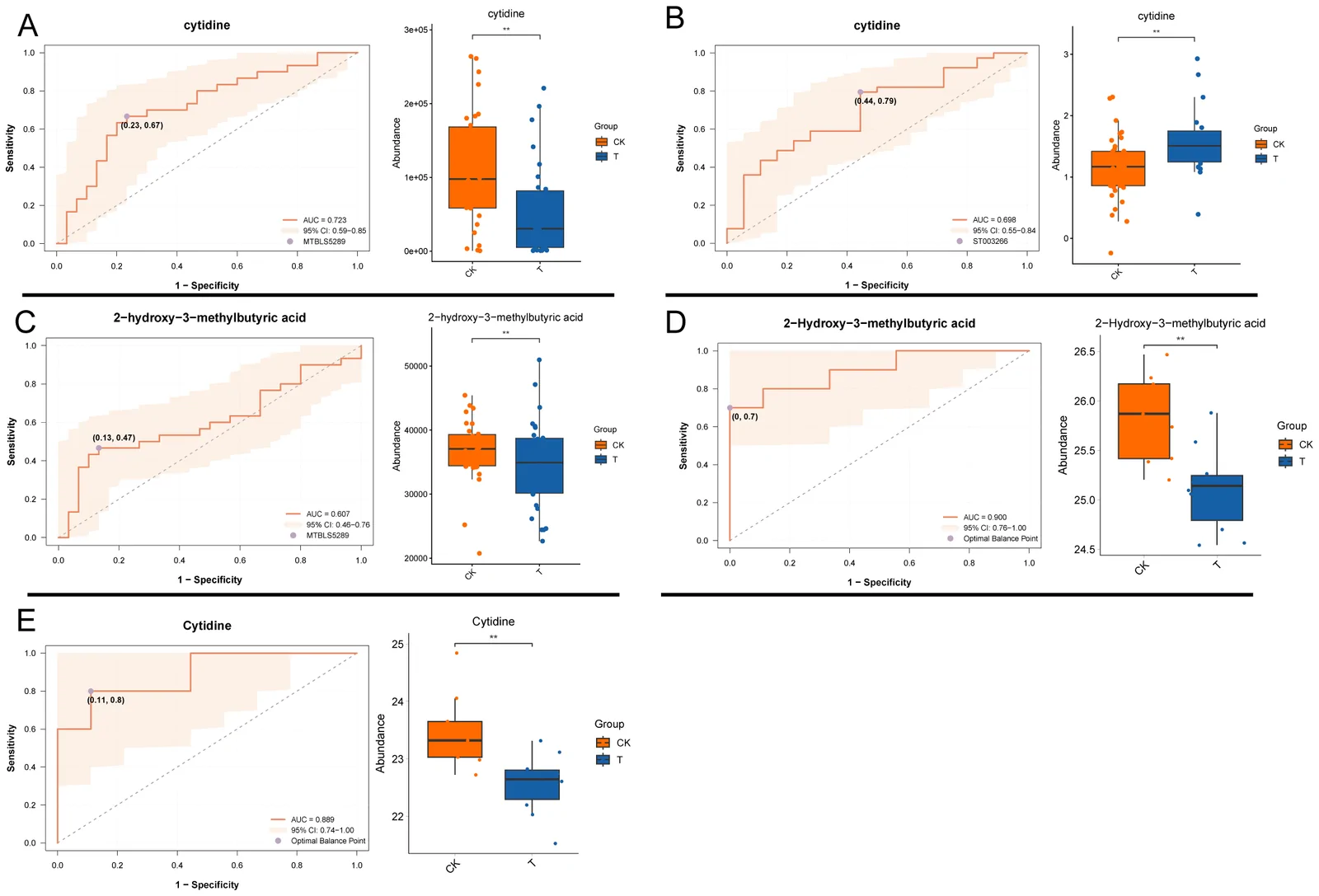
Qualitative cross-dataset comparison and *post hoc* ROC analyses of cytidine and 2-HMBA. **(A)** ROC curve and relative-abundance distribution of cytidine in MTBLS5289. **(B)** ROC curve and relative-abundance distribution of cytidine in ST003266. **(C)** ROC curve and relative-abundance distribution of 2-HMBA in MTBLS5289. **(D)** *Post hoc* ROC curve and relative-abundance distribution of 2-HMBA in the present untargeted discovery cohort. **(E)** *Post hoc* ROC curve and relative-abundance distribution of cytidine in the present untargeted discovery cohort. The ROC curves shown for the public datasets were generated *post hoc* as dataset-internal descriptive analyses. They were not interpreted as estimates of generalizable diagnostic performance and did not constitute external validation. For MTBLS5289, CK and T denote the young-semen and aged-semen groups, respectively; for ST003266, CK and T denote normozoospermia and asthenozoospermia, respectively. For the present discovery cohort, CK and T denote normozoospermic controls and men with impaired semen quality, respectively. ** indicates a nominal p value below 0.01 for the group comparison before FDR correction.

Cytidine showed lower abundance in the aged-semen group than in the young-semen group in MTBLS5289, but higher abundance in the asthenozoospermia group than in the normozoospermia group in ST003266, indicating inconsistent cross-dataset directionality (**Figures 5A, B**). In contrast, 2-HMBA showed a direction concordant between the present discovery cohort and MTBLS5289 (**Figure 5C**). It was therefore prioritized on the basis of agreement with MTBLS5289, rather than reproduction across all external datasets. Cytidine was retained for independent evaluation because it represented the discovery-level nucleotide-related pathway signal, not because of cross-dataset consistency.

### 3.8 *Post hoc* ROC analysis in the discovery cohort

*Post hoc* ROC analyses were performed to describe within-cohort group separation for 2-HMBA and cytidine. The AUCs were 0.900 (95% CI, 0.76–1.00) for 2-HMBA and 0.889 (95% CI, 0.74–1.00) for cytidine (**Figures 5D, E**). Because candidate selection and ROC evaluation were performed in the same small discovery cohort, these AUCs are likely optimistic and should not be interpreted as independent estimates of diagnostic performance.

### 3.9 Exploratory associations between candidate metabolites and semen quality parameters

To characterize associations with the multidimensional semen phenotype, Spearman correlations were calculated between the two prioritized metabolites and semen quality parameters. Benjamini–Hochberg correction was applied jointly to the p values from all correlations between the two metabolites and these parameters. Associations with age and BMI were examined separately as descriptive analyses; neither candidate showed a nominally significant association with either variable (all unadjusted p > 0.05).

**Figure 6:**
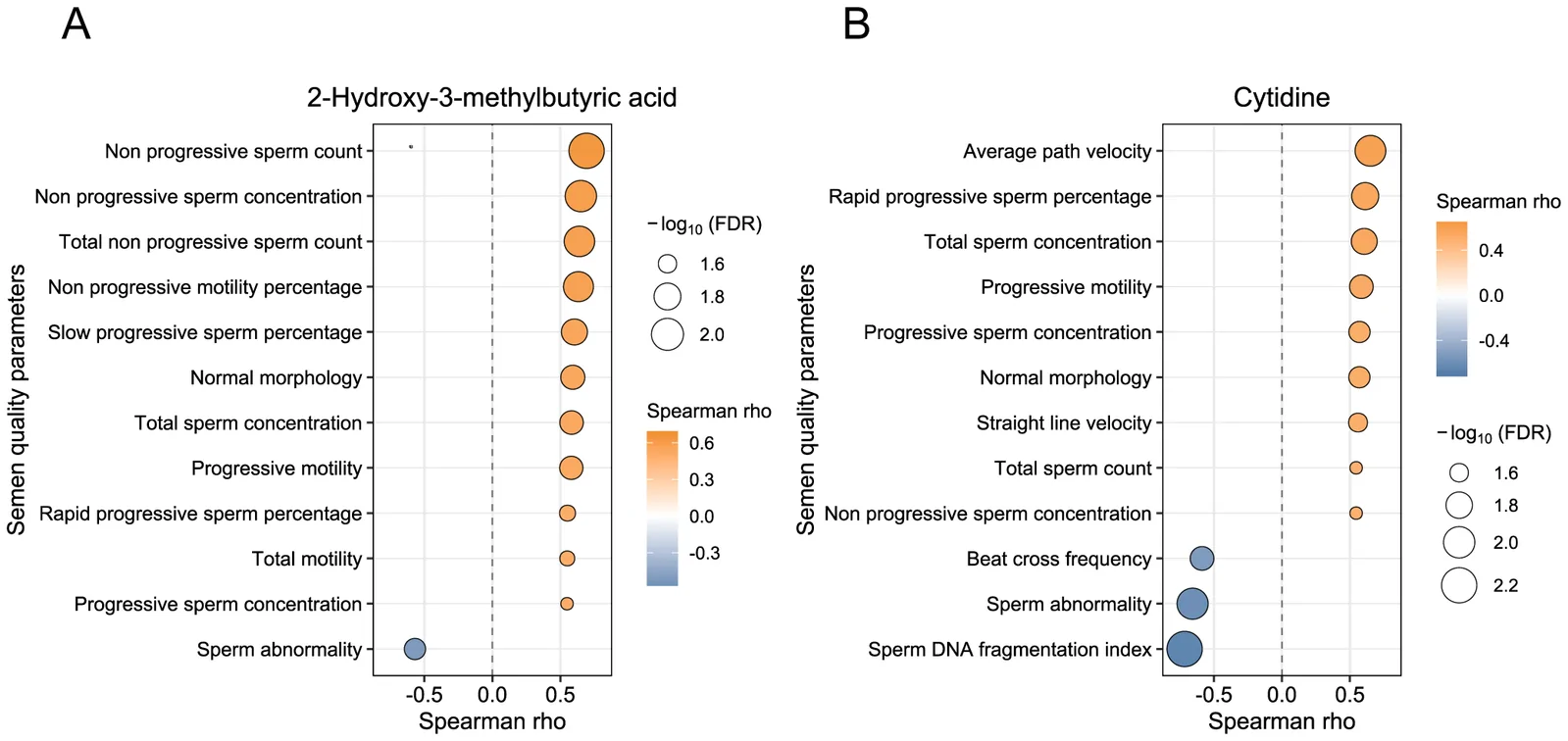
Correlation analysis of candidate metabolites with semen quality parameters. **(A, B)** Bubble plots showing Spearman correlations between the relative abundance of each prioritized candidate metabolite and conventional semen quality parameters in the untargeted discovery cohort. The horizontal position and color of each bubble represent the direction and magnitude of Spearman’s correlation coefficient (ρ), whereas bubble size represents −log10(FDR-adjusted q value). Only associations meeting an FDR-adjusted q value <0.05 are shown. The analyzed metabolites are **(A)** 2-HMBA and **(B)** cytidine.

Several metabolite–phenotype associations remained significant after FDR correction (q < 0.05). The signal assigned to 2-HMBA was positively correlated with multiple sperm count and motility measures and with normal morphology, and negatively correlated with the sperm abnormality measure (**Figure 6A**). Cytidine was positively correlated with several measures of sperm count, motility, kinematics, and morphology and negatively correlated with beat-cross frequency, the sperm abnormality measure, and sperm DNA fragmentation index (**Figure 6B**). Because candidate screening and correlation testing were conducted in the same small discovery cohort and several semen variables were biologically or mathematically interrelated, these associations provide phenotype-related context but do not establish independent or phenotype-specific metabolite–phenotype relationships.

### 3.10 Independent HPLC–UV evaluation of candidate metabolites

An independent HPLC–UV evaluation was performed in 18 men with impaired semen quality and 18 normozoospermic controls. Values are reported as external-calibration-derived estimates expressed on a nominal seminal-plasma concentration scale, as defined in the Methods, and should not be interpreted as validated quantitative concentrations. In both groups, the upper quartile for each candidate remained below the original-sample-equivalent lower calibration level, indicating that most biological-sample estimates were derived by downward extrapolation. Representative calibration, blank, and biological-sample chromatograms are provided in **Supplementary Figures 2** and **3**, and detailed calibration results are provided in **Supplementary Table 2**.

**Figure 7:**
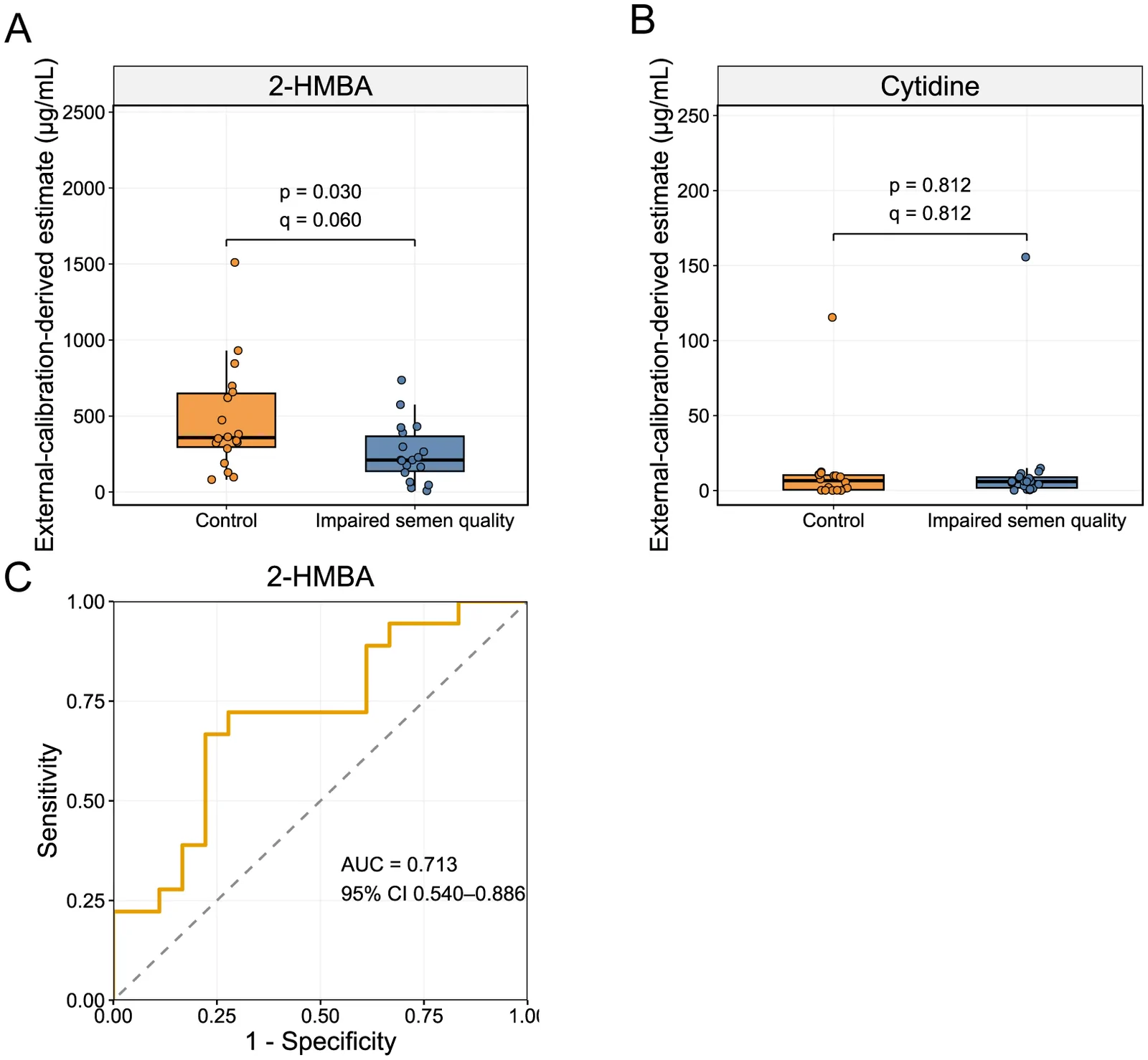
Independent evaluation of candidate metabolites using HPLC–UV analysis. **(A)** External-calibration-derived estimates assigned to 2-HMBA in normozoospermic controls and men with impaired semen quality. The estimates were nominally lower in the impaired semen quality group (Mann–Whitney U test, nominal p = 0.030) but did not remain significant after Benjamini–Hochberg correction across the two candidate comparisons (q = 0.060). **(B)** External-calibration-derived estimates assigned to cytidine in the independent cohort. No between-group difference was observed (Mann–Whitney U test, nominal p = 0.812; Benjamini–Hochberg-adjusted q = 0.812). **(C)** Exploratory *post hoc* receiver operating characteristic (ROC) analysis based on estimates assigned to 2-HMBA. The area under the curve (AUC) was 0.713 (95% confidence interval [CI], 0.540–0.886). The ROC analysis was not interpreted as evidence of diagnostic performance or biomarker utility. Values were expressed on a nominal seminal-plasma concentration scale using external calibration. In both groups, the upper quartile for each candidate was below the original-sample-equivalent lower calibration level, indicating that most estimates were derived by downward extrapolation and were not measurements within a validated quantitative range. Individual points represent participants. Boxes indicate the interquartile range, center lines indicate medians, and whiskers represent 1.5 × interquartile range.

The median external-calibration-derived estimate assigned to 2-HMBA was 358 (295, 649) μg/mL in controls and 211 (137, 366) μg/mL in the impaired semen quality group (**Figure 7A**). The estimates were nominally lower in the impaired semen quality group (Mann–Whitney U test, p = 0.030), but the difference did not remain significant after correction across the two HPLC–UV candidate comparisons (q = 0.060). The direction was consistent with the untargeted discovery observation, but this directional agreement did not constitute quantitative or analyte-specific validation.

The median external-calibration-derived estimate assigned to cytidine was 6.59 (0.556, 10.3) μg/mL in controls and 5.99 (1.82, 8.90) μg/mL in the impaired semen quality group (**Figure 7B**). No between-group difference was observed (p = 0.812; q = 0.812), and the discovery-level cytidine alteration was therefore not reproduced in the independent cohort.

*Post hoc* ROC analysis of 2-HMBA yielded an AUC of 0.713 (95% CI, 0.540–0.886) (**Figure 7C**). The moderate AUC and wide confidence interval indicated imprecise within-cohort discrimination, and no prespecified clinical threshold was evaluated.

Overall, the independent HPLC–UV evaluation provided a nominally lower, directionally consistent signal assigned to 2-HMBA that was not significant after correction, whereas cytidine showed no independent between-group difference. These findings were treated as candidate-prioritization evidence only.

## 4 Discussion

In this exploratory study, untargeted seminal plasma metabolomics was used to characterize metabolic alterations associated with impaired semen quality, followed by qualitative cross-dataset comparison and independent HPLC–UV evaluation of selected candidates. No individual metabolite remained significant after Benjamini–Hochberg correction, and the limited cross-validated performance and permutation-test results did not support a robust OPLS–DA model. The findings should therefore be considered hypothesis-generating and interpreted as two distinct lines of evidence. The first was a pathway-level signal involving nucleotide- and nucleoside-related processes; cytidine was selected as a representative candidate but showed no between-group difference in the independent cohort. The second was an individual signal assigned to 2-HMBA, which showed directional consistency across the available comparisons but did not achieve FDR-adjusted significance in the independent evaluation. Neither line of evidence establishes a specific biological mechanism or supports the clinical use of either metabolite as a biomarker.

The multidimensional phenotype of the affected group is central to interpreting these findings. Although reduced sperm motility was a defining feature, affected participants also had abnormal morphology, lower sperm counts and total progressive sperm counts, and greater sperm DNA fragmentation. The observed metabolic alterations therefore cannot be attributed specifically to impaired motility or interpreted as an asthenozoospermia-specific signature. Moreover, seminal plasma is a heterogeneous fluid containing contributions from the testes, epididymis, prostate, seminal vesicles, and other components of the reproductive tract. Changes in seminal plasma metabolite abundance may therefore reflect differences in secretion, transport, cellular turnover, or the relative contributions of reproductive tract fluids rather than alterations in sperm-intrinsic metabolism alone (25).

Consistent with this multidimensional phenotype, exploratory analyses linked 2-HMBA to sperm count, motility, and morphology-related measures, whereas cytidine was associated with sperm count, motility and kinematic measures, morphology-related measures, and sperm DNA fragmentation. Previous studies have likewise reported metabolic alterations in spermatozoa from men with idiopathic asthenozoospermia and in seminal plasma from men with abnormal sperm morphology (26, 27), supporting the broader concept that metabolic disturbances may accompany different forms of impaired semen quality. However, the associations observed here do not establish phenotype specificity because candidate selection and correlation testing were conducted in the same small discovery cohort, several semen variables were biologically or mathematically interrelated, and the available studies do not demonstrate that the same metabolites or mechanisms are shared across motility, morphology, and combined semen abnormalities.

The first line of evidence was a pathway-level signal involving pyrimidine metabolism, nucleotide recycling, and nucleoside transport. Pyrimidine nucleotides contribute to nucleic acid synthesis and serve as activated intermediates in several cellular biosynthetic processes (28). In seminal plasma, however, changes in cytidine and other nucleoside-related metabolites may reflect multiple intracellular and extracellular processes, including nucleotide turnover, cellular release, and secretions from different regions of the reproductive tract. Although sperm function depends on coordinated glycolytic and mitochondrial ATP production, and mitochondrial status has been associated with motility and other semen characteristics (29, 30), these relationships provide only biological context for the present findings. Pyrimidine metabolism cannot be equated with ATP production, and this study did not directly assess intracellular nucleotide pools, adenylate charge, mitochondrial respiration, or related functional measures. Moreover, KEGG, Reactome, and IPA were applied to the same nominally prioritized metabolite set and therefore do not represent independent replication. Given the additional influence of metabolite annotation, background-set selection, database coverage, and analytical method on pathway enrichment (18), the nucleotide-related signal should be considered a testable hypothesis rather than evidence of established nucleotide or bioenergetic dysfunction.

Within this pathway-level signal, cytidine was selected as a representative candidate rather than as a metabolite that showed consistent behavior across datasets. Its direction of change varied among the public datasets, and cytidine did not differ between groups in the independent HPLC–UV cohort. These findings do not support cytidine as an individually reproducible candidate. The pathway-level signal may instead reflect coordinated but modest changes across several nucleotide-related metabolites rather than a dominant alteration in a single compound. Differences in biological matrix, extracellular nucleotide turnover, analytical platform, preprocessing, and annotation may also have contributed to the inconsistent findings. Thus, the lack of replication weakens the evidence for a cytidine-specific alteration, although it does not, by itself, exclude the broader hypothesis of altered nucleotide-related processes.

The second line of evidence concerned the signal assigned to 2-HMBA, which showed the most consistent directional pattern among the prioritized individual candidates. Also referred to as 2-hydroxyisovaleric acid, 2-HMBA has been linked to branched-chain amino acid (BCAA) metabolism. BCAAs and their catabolic intermediates participate in cellular energy metabolism and metabolic signaling (31). Experimental evidence from mice has linked the regulation of BCAA metabolism in the testes and sperm to energy homeostasis and sperm migration, although the reported mechanism was PRSS55-dependent and cannot be directly extrapolated to the present human cohort (32). In humans, branched-chain and aromatic amino acids measured in blood and seminal plasma have also been associated with sperm parameters, with systemic metabolic status representing a potentially important influence (33). Nevertheless, the biological meaning of a lower seminal plasma 2-HMBA signal remains uncertain. Metabolite abundance reflects the balance among production, utilization, transport, and clearance, as well as contributions from different reproductive tract secretions. The present findings therefore do not demonstrate reduced BCAA catabolism or establish that lower 2-HMBA contributes to impaired mitochondrial function, ATP production, sperm morphology, or motility.

Despite this uncertainty, the signal assigned to 2-HMBA remained of interest because its direction was consistent across the available comparisons: its annotated abundance was lower in the discovery cohort, the available MTBLS5289 comparison showed the same direction, and the HPLC–UV response-derived estimates were lower in the independent cohort. However, directional consistency does not constitute statistical replication, analyte-specific confirmation, or quantitative validation. In the independent cohort, the difference was nominally significant but did not remain significant after correction for the two candidate comparisons (p = 0.030; q = 0.060). Moreover, most HPLC–UV estimates were extrapolated below the lowest calibration level, and the assay lacked an internal standard, sample-level confirmatory MS/MS, and comprehensive validation of precision, recovery, matrix effects, and quantification limits. The *post hoc* AUC of 0.713, with a wide 95% confidence interval of 0.540–0.886, indicated only moderate and imprecise within-cohort discrimination. Given the small sample size, *post hoc* analysis, and absence of a prespecified classification threshold, these findings support further analytical investigation of the 2-HMBA assigned signal but do not support its interpretation as a validated metabolite alteration or clinical biomarker (34).

The strengths of this study include the use of separate cohorts for untargeted discovery and independent HPLC–UV evaluation, the use of authentic reference standards, transparent reporting of multiple-testing and OPLS–DA results, careful consideration of heterogeneity among the external datasets, and standardized preanalytical handling with pooled-QC monitoring of the untargeted LC–MS/MS analysis.

Several limitations should be acknowledged. First, the small sample sizes of both the untargeted discovery cohort and the independent HPLC–UV cohort substantially limited statistical power, precision, and robustness and precluded reliable multivariable adjustment and phenotype-stratified analyses. No individual metabolite remained significant after FDR correction, and the OPLS–DA model showed limited cross-validated performance and was not supported by the permutation-test diagnostics. The candidate-metabolite, pathway, correlation, and *post hoc* ROC findings should therefore be considered hypothesis-generating. In particular, the discovery-cohort AUC estimates may be optimistic because candidate selection and performance assessment were conducted in the same participants (34). The cross-sectional design also prevents conclusions about whether the observed metabolic differences preceded, accompanied, or resulted from impaired semen quality. In addition, several semen variables were biologically or mathematically interrelated, and the metabolite–phenotype correlations were calculated across the pooled case–control sample after candidate selection based partly on between-group differences. Some associations may therefore reflect case–control separation or overlapping aspects of the same phenotype rather than independent biological relationships. The small within-group sample sizes precluded reliable stratified or group-adjusted correlation analyses.

Second, the affected group had multidimensional impairment in semen quality rather than isolated asthenozoospermia. The study design therefore cannot determine whether the observed metabolic signals were associated primarily with impaired motility, abnormal morphology, reduced sperm number, increased DNA fragmentation, or biological factors shared across these abnormalities. In addition, detailed information on smoking, alcohol consumption, dietary habits, medication use, metabolic syndrome–related factors, leukocytospermia, and inflammatory status was not systematically collected. Residual and unmeasured confounding therefore cannot be excluded.

Third, although processing time, storage conditions, storage duration, and freeze–thaw exposure were standardized, the single pre-storage centrifugation step may not have completely removed residual spermatozoa, leukocytes, epithelial cells, or cellular debris. Seminal plasma also contains extracellular vesicles originating from multiple regions of the male reproductive tract (35). The measured metabolome may therefore reflect a mixture of cellular, extracellular-vesicle, and reproductive tract secretory components rather than a strictly cell-free extracellular metabolic profile.

Fourth, the external datasets provided only qualitative context because of substantial differences in biological matrix, phenotype definition, analytical platform, preprocessing, and metabolite annotation. The HPLC–UV method also lacked an internal standard, pooled targeted quality-control samples, sample-level confirmatory MS/MS, and comprehensive validation of precision, recovery, matrix effects, and quantification limits. Most biological-sample estimates were extrapolated below the lowest calibration level. In addition, although peaks were assigned using analyte-specific retention-time regions and authentic reference standards, the single-wavelength method and absence of formal peak-purity assessment could not exclude contributions from coeluting matrix components. The HPLC–UV results should therefore be interpreted as exploratory response-derived signals assigned to the candidate analytes rather than as definitive analyte-specific concentrations.

Future studies should first evaluate the reproducibility and phenotype specificity of these findings in prospectively powered, multicenter cohorts with prespecified primary endpoints. Recruitment should include sufficiently large groups representing isolated motility impairment, isolated morphological abnormalities, reduced sperm number, combined semen abnormalities, and normozoospermic controls. Such stratification would help determine whether the observed metabolic signals are associated specifically with individual semen phenotypes or instead reflect biological processes shared across broader impairment in semen quality. Repeated semen and seminal plasma collection would also allow assessment of within-individual stability and reduce the influence of normal temporal variation. Lifestyle, dietary, medication-related, metabolic, inflammatory, and reproductive tract factors should be systematically recorded and incorporated into appropriately powered multivariable analyses. Candidate metabolites should be measured in independent replication cohorts using analytically validated quantitative methods, preferably isotope-dilution LC–MS/MS with stable-isotope-labeled internal standards, matrix-matched calibration, and predefined quality-control procedures. Validation should include assessments of selectivity, linearity, precision, accuracy, recovery, matrix effects, stability, and limits of detection and quantification.

Mechanistic studies should analyze paired seminal plasma and sperm samples to determine whether nucleotide- and BCAA-related signals reflect sperm-intrinsic metabolism, accessory gland secretion, systemic metabolic status, or other components of the seminal plasma microenvironment. Targeted measurement of nucleotides, nucleosides, BCAAs, and related catabolic intermediates should be integrated with direct assessments of ATP, ADP, AMP, adenylate charge, NAD^+^/NADH balance, mitochondrial respiration, membrane potential, reactive oxygen species, and antioxidant capacity. Where feasible, isotope-tracing experiments could be used to examine metabolic flux through nucleotide recycling and BCAA catabolism rather than relying solely on static metabolite concentrations. Functional experiments exposing human spermatozoa or suitable experimental models to physiologically relevant concentrations of prioritized metabolites could further assess effects on motility, mitochondrial activity, oxidative stress, capacitation, acrosome reaction, and fertilization-related functions. Ultimately, the clinical relevance of these signals should be evaluated only after independent analytical replication and biological confirmation, followed by prospective assessment of their associations with clinically meaningful reproductive outcomes, such as fertilization, embryo development, pregnancy, and live birth.

## 5 Conclusion

This exploratory study identified seminal-plasma metabolic signals associated with multiple concurrent semen abnormalities. The small sample sizes in both cohorts limited statistical power, precision, and robustness. The OPLS–DA model showed limited cross-validated predictive performance and was not considered a robust predictive classifier. No individual metabolite remained significant after FDR correction in the discovery analysis. The nucleotide- and nucleoside-related pathway signal therefore remains hypothesis-generating and requires confirmation in independent, adequately powered cohorts together with direct functional investigation. In the independent HPLC–UV evaluation, the signal assigned to 2-HMBA was nominally lower and directionally consistent with the discovery observation, but the difference did not remain significant after correction; cytidine showed no between-group difference. Because most HPLC–UV estimates were extrapolated below the lowest calibration level and the assay lacked analyte-specific confirmation and comprehensive quantitative validation, these results support only further investigation of the signal assigned to 2-HMBA. They neither establish a specific metabolic mechanism nor support the current use of 2-HMBA as a clinical biomarker.

## Data Availability

The datasets presented in this study can be found in online repositories. The names of the repository/repositories and accession number(s) can be found in the article/**Supplementary Material**.
