# Supplementary material for: Exploratory seminal plasma metabolomics and independent HPLC–UV evaluation in men with impaired semen quality
Source: Front Endocrinol (Lausanne). 2026 Aug 25;17:1909718. doi: 10.3389/fendo.2026.1909718 (PMC13549830; doi:10.3389/fendo.2026.1909718)
Supplement: Supplementary file 1 [file SupplementaryFile1.docx]

***Supplementary Material***

**Supplementary Figures**

 **Supplementary Figure 1. Global correlation analysis of the 89 exploratory candidate metabolites.**
**(A)** Complete Spearman correlation matrix. Colors indicate correlation direction and magnitude; asterisks denote nominal p values (*p <0.05, **p <0.01, and ***p <0.001).
**(B)** Network displaying the 250 strongest correlations among the 302 pairs that remained significant at q ≤0.05 after Benjamini–Hochberg correction across all 3,916 tests. The 302 significant pairs involved 75 metabolites. Nodes represent metabolites, node colors indicate metabolite superclass, and edge colors indicate correlation direction. The 250-edge limit was used only to improve visual clarity.**Supplementary Figure 2. Analytical characteristics of the external-calibration-based HPLC–UV evaluation of cytidine and 2-hydroxy-3-methylbutyric acid (2-HMBA).**
**(A)** Representative chromatogram of the mixed CAL-3 standard containing cytidine at 60 μg/mL and 2-HMBA at 600 μg/mL, with retention times of 4.795 and 8.387 min, respectively.

**(D)** Representative solvent blank chromatogram. No reportable integrated peak was detected in the blank at the target retention-time regions. Calibration was performed using unweighted linear regression constrained through the origin.

**(B)** External calibration curve for cytidine.

**(C)** External calibration curve for 2-HMBA.

**Supplementary Figure 3. Representative HPLC–UV chromatographic regions of signals assigned to cytidine and 2-hydroxy-3-methylbutyric acid (2-HMBA) in seminal plasma samples.** Representative chromatographic regions are shown for cytidine in a normozoospermic control sample (A) and a sample from a man with impaired semen quality (B), and for 2-HMBA in a normozoospermic control sample (C) and a sample from a man with impaired semen quality (D). Dashed lines indicate the MassHunter-reported retention times, and the shaded regions indicate manually reconstructed chromatographic regions corresponding to the reported responses. The chromatograms are illustrative examples and were not used independently to infer between-group differences.

**Supplementary Tables**

**Supplementary Table 1. Characteristics of the present untargeted discovery cohort and publicly available reproductive metabolomics datasets used for qualitative cross-dataset comparison.**

**Supplementary Table 2A. Calibration characteristics of cytidine and 2-HMBA in the independent HPLC–UV evaluation.**

**Supplementary Table 2B. Calibration characteristics and point-level calibration results for the HPLC–UV evaluation.**
